# The New Sydenham Society

**Published:** 1861-11

**Authors:** 


THE NEW SYDENHAM SOCIETY.—The following works will (should nothing unforeseen prevent) be issued during the current year:—

THE SERIES FOR 1861.

I. A YEAR-BOOK OF MEDICINE AND SURGERY OF 1860. (Already out.)
II. A volume of selected monographs, comprising:—
  1. CZERMAK ON THE PRACTICAL USES OF THE LARYNGOSCOPE.
  2. SCHRŒDER VAN DER KOLK ON ATROPHY OF THE BRAIN.
  3. DUSCH ON DISEASES OF THE CEREBRAL SINUSES.
  4. ESMARCH ON THE USES OF COLD IN SURGICAL PRACTICE.
  5. RADICKE'S PAPERS ON THE APPLICATION OF STATISTICS TO MEDICAL ENQUIRIES.
III. The first volume of CASPER'S FORENSIC MEDICINE.
IV. The second and concluding volume of FRERICHS'S CLINICAL ACCOUNT OF DISEASES OF THE LIVER. (The second volume of this work has not yet been published in the original. By the courtesy of its author and publishers, (who have supplied the sheets as printed off,) the Council will be enabled to have the translation ready almost simultaneously with the appearance of the work itself.)
V. A SECOND FASCICULUS OF THE ATLAS OF PORTRAITS OF SKIN DISEASES, comprising Three Plates, illustrating
  PSORIASIS DIFFUSA.
  ICHTHYOSIS.
  LUPUS SERPIGINOSUS.
  ALOPÆCIA AREATA.

The subscription is Five Dollars and a Quarter, annually, to be paid in advance. Those members who have not yet paid their subscriptions for the current year are requested to do so as early as convenient.

Those desirous of joining the Society and of obtaining the works from the commencement, are requested to make early application. A list of the eleven works already published, with a report and other information, will be forwarded on application.

The publication of Vogel and Neubauer's work on the Examination of the Urine is deferred for a short time, in consequence of the expected appearance of a new edition of the original.

RICHARD J. DUNGLISON, M.D., 121 S. 10th St., is the Hon. Local Secretary for Philadelphia.

